# Destabilizing Protein Polymorphisms in the Genetic Background Direct Phenotypic Expression of Mutant SOD1 Toxicity

**DOI:** 10.1371/journal.pgen.1000399

**Published:** 2009-03-06

**Authors:** Tali Gidalevitz, Thomas Krupinski, Susana Garcia, Richard I. Morimoto

**Affiliations:** Department of Biochemistry, Molecular Biology, and Cell Biology, Rice Institute for Biomedical Research, Northwestern University, Evanston, Illinois, United States of America; University of Minnesota, United States of America

## Abstract

Genetic background exerts a strong modulatory effect on the toxicity of aggregation-prone proteins in conformational diseases. In addition to influencing the misfolding and aggregation behavior of the mutant proteins, polymorphisms in putative modifier genes may affect the molecular processes leading to the disease phenotype. Mutations in *SOD1* in a subset of familial amyotrophic lateral sclerosis (ALS) cases confer dominant but clinically variable toxicity, thought to be mediated by misfolding and aggregation of mutant SOD1 protein. While the mechanism of toxicity remains unknown, both the nature of the *SOD1* mutation and the genetic background in which it is expressed appear important. To address this, we established a *Caenorhabditis elegans* model to systematically examine the aggregation behavior and genetic interactions of mutant forms of SOD1. Expression of three structurally distinct SOD1 mutants in *C. elegans* muscle cells resulted in the appearance of heterogeneous populations of aggregates and was associated with only mild cellular dysfunction. However, introduction of destabilizing temperature-sensitive mutations into the genetic background strongly enhanced the toxicity of SOD1 mutants, resulting in exposure of several deleterious phenotypes at permissive conditions in a manner dependent on the specific SOD1 mutation. The nature of the observed phenotype was dependent on the temperature-sensitive mutation present, while its penetrance reflected the specific combination of temperature-sensitive and SOD1 mutations. Thus, the specific toxic phenotypes of conformational disease may not be simply due to misfolding/aggregation toxicity of the causative mutant proteins, but may be defined by their genetic interactions with cellular pathways harboring mildly destabilizing missense alleles.

## Author Summary

Correct folding and stability are essential for protein function. In cells, a network of molecular chaperones and degradative enzymes facilitate folding, prevent aggregation and ensure degradation of the misfolded proteins, thus maintaining protein homeostasis. In many diseases, including Amyotrophic Lateral Sclerosis (ALS), expression of a single mutant protein that misfolds and aggregates causes cellular toxicity that is strongly dependent on the genetic background. To address the influence of genetic background on the toxicity of aggregation-prone proteins, we established a *C. elegans* model of misfolding and aggregation of several distinct ALS-related mutants of superoxide dismutase 1 (SOD1). In one wild type genetic background (N2), these proteins exhibited only mild cellular toxicity despite strong, mutant-specific aggregation phenotypes. However, when SOD1 mutants were expressed in the background of mildly destabilized protein polymorphisms, their toxicity was enhanced and a number of distinct phenotypes were exposed. These synthetic phenotypes reflected the loss-of-function of the destabilized polymorphic proteins. Furthermore, the degree to which each of these phenotypes was exposed depended on the nature of the SOD1 mutation. These data suggest that the presence of mildly destabilizing polymorphisms in the genetic background may modulate and direct the specific toxic phenotypes in protein aggregation diseases.

## Introduction

ALS (OMIM #105400 http://www.ncbi.nlm.nih.gov/entrez/dispomim.cgi?cmd=entry&id=105400) is a progressive degenerative disorder affecting motor neurons in the brain stem and spinal cord. Up to 10% of cases have a dominant familial inheritance pattern with mutations in *SOD1* (OMIM *14750 http://www.ncbi.nlm.nih.gov/entrez/dispomim.cgi?cmd=entry&id=147450) contributing about 20% of those [1],[2]. While it is accepted that disease results from toxic gain of function by the mutant protein [3]–[5], the mechanisms contributing to toxicity remain unknown. Two main hypotheses have been proposed; the first invokes abnormal chemistry of mutant SOD1 proteins, resulting in nitration of tyrosine residues on cellular proteins [6] and increased production of hydroxyl radicals [7],[8]. However, mutant SOD1 retains its toxic properties even when abnormal chemical reactions are greatly reduced [9] suggesting that abnormal chemistry alone may not be the basis of toxicity. Furthermore, the role of the dismutase genes in preventing the long-term protein damage have recently being questioned [10]. The second hypothesis suggests that, as for many other neurodegenerative diseases, the toxicity is mediated by misfolding and aggregation of mutant proteins [9], [11]–[13]. Accumulation of proteinaceous inclusions in conformational disease indicates an inability of the protein folding quality control machinery to efficiently recognize, fold, and degrade abnormal proteins [14], including the mutant forms of SOD1. The role of damaged proteins is further supported by observations that elevated levels of molecular chaperones decrease mutant SOD1 toxicity [15],[16]. However, it is still unclear how misfolding or aggregation of SOD1 mutant protein leads to cellular toxicity.

ALS patients harboring different or even the same *SOD1* mutations exhibit a high degree of clinicopathologic variation, including clinical severity, age at onset, and the types of motor neurons involved [17]–[20]. Both different biophysical properties of mutant proteins and variation in the genetic background may independently modulate the toxicity, providing a range of phenotypes [21],[22]. The importance of genetic interactions in modulating disease is further underscored by findings that ALS phenotypes in transgenic mice vary greatly depending on the strain in which the mutant protein is expressed [23],[24]. Understanding the differences between SOD1 mutants in misfolding/aggregation behavior and in their interactions with cellular proteins and pathways may thus provide insights into the toxic mechanisms and the nature of modifier genes.

To systematically examine the aggregation behavior and genetic interactions of mutant forms of SOD1, we established a *C. elegans* model expressing human SOD1-YFP fusion proteins in the body-wall muscle cells. The ability to employ dynamic imaging in live animals throughout their lifespan and availability of both forward and reverse genetic approaches makes *C. elegans* an attractive model to study aggregation toxicity. Similar models in *C. elegans* have been used to investigate the aggregation toxicity and genetic modifiers of polyglutamine expansions and α-synuclein [25]–[27]. Here, we show that three biophysically distinct [28] mutants of SOD1 form strikingly polymorphic aggregates in *C. elegans*. Expression of mutant SOD1 alone was associated with mild toxicity. However, when mutant SOD1 was introduced into genetic backgrounds harboring destabilizing temperature-sensitive mutations, the toxicity was enhanced significantly and a variety of toxic phenotypes was observed. These phenotypes reflected both the specific SOD1 mutant and the loss-of-function of each of the destabilized temperature-sensitive proteins. Thus, we propose that specific phenotypes in conformational disease may be influenced by the mildly destabilizing missense mutations present in the genetic background.

## Results

### *C. elegans* Model for Expression of Wild Type and Mutant Human SOD1-YFP Fusions

We established a *C. elegans* model to study SOD1 aggregation toxicity by expressing wild type and mutant SOD1 in body wall muscle cells, employing a tissue-specific promoter (p*Unc-54*) and C-terminal YFP-tagging scheme (Figure S1*A*) [25]. The YFP-tagged wild type SOD1 retained its enzymatic activity (Figure S2, lane 1), indicating that the tag does not interfere with SOD1 folding. Because various mutations in the SOD1 protein exhibit different biophysical and biochemical properties [28], we chose three distinct mutant SOD1 proteins associated with ALS. G85R is representative of inactive “metal-binding” mutants [29], deficient in copper and zinc binding and significantly destabilized [30]. G93A represents “wild type-like” mutants that bind copper and zinc, exhibit mild loss of thermal stability when fully metallated, and retain enzymatic activity [31]. 127X (G127insTGGGstop) is a frameshift mutation resulting in a C-terminal truncation of the last 21 amino acids and a highly unstable protein [32]. These mutants form protein aggregates with toxic phenotypes when expressed in mammalian cultured cells and transgenic mice [3],[5],[32],[33].

Transgenic lines expressing p*Unc-54*::WT SOD1::YFP ( WT SOD1), p*Unc-54*::SOD1-G85R::YFP (G85R), p*Unc-54*::SOD1-G93A::YFP (G93A) and p*Unc-54*::SOD1- G127insTGGGstop::YFP (127X) were established. We verified that SOD1 proteins expressed in the muscle cells were of the expected molecular sizes (Figure S1*B*). Only transgenic lines expressing steady-state levels of mutant proteins similar to or lower than WT SOD1 were selected for further study since high expression levels could influence aggregation and toxicity (Figure S1*B*).

**Figure 1:**
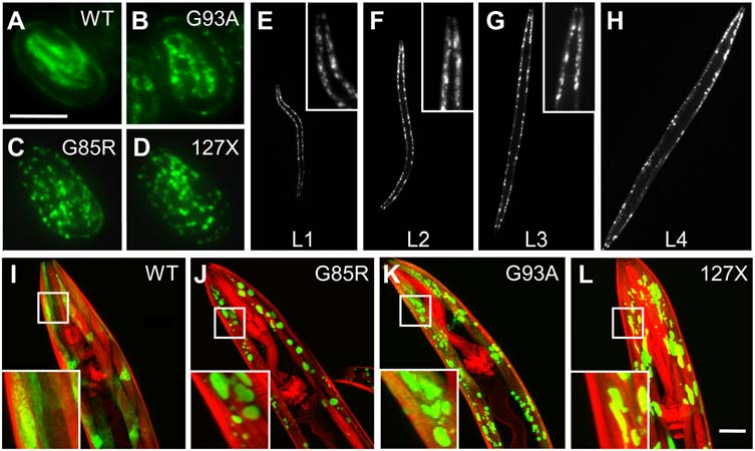
SOD1 mutant proteins aggregate in the body wall muscle cells of *C. elegans*. (*A–D*) Fluorescent micrographs of three-fold embryos showing the diffuse pattern of WT SOD1-YFP fusion (*A*) and punctate fluorescence of YFP fusions of G93A (*B*), G85R (*C*) and 127X (*D*). (*E–H*) G85R animals in all larval stages – L1 (*E*), L2 (*F*), L3 (*G*), L4 (*H*) – display punctate fluorescent pattern. Inserts are close-ups of the head area. (*I–L*) Confocal projections of transgenic adult animals showing the distribution of the SOD-YFP fluorescence (green) and Rhodamine-phalloidin stained myofilaments (red). WT SOD1 protein exhibits diffuse, if patchy, fluorescence (*I*), while all mutant strains contain discrete fluorescent foci as well as some diffuse fluorescence (*J* through *L*). Inserts are close-ups of the boxed areas. Scale bars in *A* and *L* are 50 and 20 micrometers, respectively. All images are of representative animals with typical fluorescent patterns.

**Figure 2:**
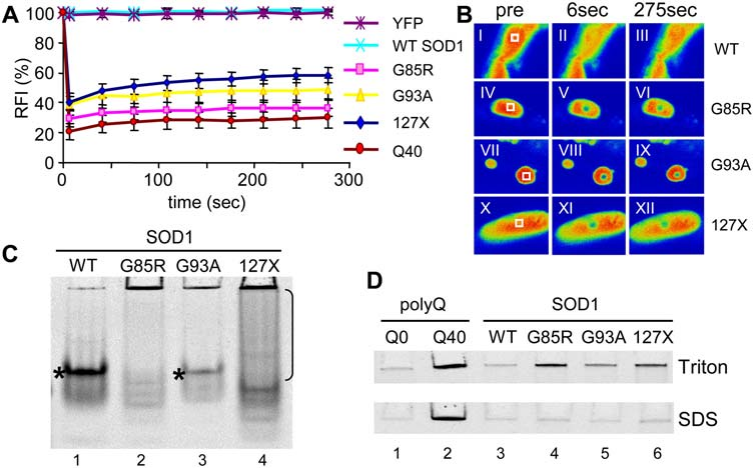
Mutant SOD1 proteins form biophysically variable aggregates. (*A*) FRAP analysis of fluorescent foci of SOD1. WT SOD1 protein (light blue) is indistinguishable from soluble YFP protein (purple). The fluorescence recovery of G85R and G93A (pink and yellow) is consistent with stable aggregates, but shows higher initial recovery than polyQ40 species (red). 127X protein demonstrates continued slow recovery consistent with less stable aggregates (blue). The traces are averages of 7 replicates. (*B*) Fluorescent micrographs of representative FRAP experiments. Panels I through III show a concentrated area of fluorescence in WT SOD1 muscle cell before bleaching (pre), immediately post bleaching (6 seconds) and at the end of the experiment (275 seconds). The bleached area is denoted by a white square. Panels IV through XII show FRAP experiments on G85R (IV to VI), G93A (VII to IX) and 127X (X to XII) foci, note presence of bleached area even at 275 seconds post bleach. (*C*). 7.5% native PAGE of extracts from indicated mutant strains. Mutant proteins contain high molecular weight material that did not enter the gel (lanes 2 to 4). WT SOD1 (lane 1) and G93A (lane 3) contain a major soluble band (star), which corresponds to enzymatically active SOD1 protein (Figure S2, arrows). G85R (lane 2) and 127X (lane 4) exhibit presence of multiple soluble species, none of which contain enzymatic activity (Figure 2S); 127X also presents unresolved smear below the aggregated material (bracket), suggesting a continual dissociation of aggregates during electrophoresis. (*D*) Native extracts were treated with 0.5% Triton X-100 or 5% SDS for 15 minutes at room temperature and resolved by 5% native PAGE. SOD1 mutants contained aggregates that dissociated upon SDS treatment (lower panel, lines 4 to 6). Polyglutamine aggregates were used for comparison (line 2). Gels in panels (*C*) and (*D*) are representative of at least three experiments.

Wild type SOD1 exhibited diffuse fluorescence in body wall muscle cells throughout development and during adulthood (Figure 1*A,I*) with broad distribution in the muscle belly (the cytoplasmic space of a muscle cell below the myofilaments) and the muscle arms (the projections from muscle cells toward the neural ring). Although WT SOD1 had patchy appearance in some of the cells, the brighter areas were diffuse upon examination at higher magnification (insert in Figure 1*I*), corresponding to soluble protein (Figure 2*A*). In contrast, all three mutant SOD1 proteins presented a punctate fluorescent pattern that appeared in embryonic stages (Figure 1*B–D*) and persisted throughout larval development and adulthood (Figure 1 and Figure S3). In all three mutant forms of SOD1, we observed both diffuse and punctate fluorescence corresponding to two populations of protein. Thus, SOD1 proteins in *C. elegans* exhibit properties similar to those observed in other model systems, where only the mutant SOD1 protein forms inclusions when expressed ectopically [3],[5],[32],[33].

### Mutant SOD1 Forms Biophysically Distinct Classes of Protein Aggregates

Accumulation of mutant SOD1 proteins into visible foci, although consistent with their *in vitro* aggregation propensity, does not necessarily indicate the formation of aggregates. We used dynamic imaging and FRAP analysis to establish the aggregation state of SOD1 proteins in live animals. As shown in Figure 2*A*, the diffusion of WT SOD1-YFP fusion protein in body wall muscle cells (light blue) was indistinguishable from that of YFP alone (purple), with nearly complete recovery within the dead-time of measurement post bleaching. In contrast, the fluorescent foci of all three mutant proteins exhibited reduced recovery indicative of immobile aggregate species (Figure 2*A,B*). G85R and G93A proteins had 35 and 50% recovery over 275 seconds, respectively, higher than that observed for foci of well-characterized aggregation-prone polyQ40 (30%), which contain only an immobile protein [34]. The recovery of fluorescence in 127X foci continued beyond 100 sec and reached nearly 60% over the course of the experiment, which suggests either partially mobile species, or the presence of multiple populations of protein.

Since these SOD1 mutants have different structural and biophysical properties *in vitro* [28], the observed differences in the fluorescence recovery of aggregates could reflect the presence of different molecular species or interactions *in vivo*. We analyzed the oligomeric state of SOD1 proteins by native gel electrophoresis. Extracts from G85R, G93A and 127X lines contained soluble SOD1 protein in addition to large aggregate species that did not enter the gel, while extracts of WT SOD1 lines contained mainly soluble protein (Figure 2*C*). The distribution of the soluble G93A protein appeared similar to the WT SOD1, with one major band containing enzymatically active protein (Figures 2*C*, star and S2, arrow). In contrast, G85R and 127X were resolved as multiple species of different intensities lacking enzymatic activity (Figures 2*C* and S2*B*). This is in agreement with the known native-like properties of G93A [31] and suggests that human SOD1 proteins can preserve their characteristics when expressed in *C. elegans*. 127X extracts also contained a heterogeneous population of electrophoretic states (Figure 2*C*, bracket), which could indicate conformational instability and continual dissociation of larger molecular species, in agreement with the continual recovery of fluorescence by FRAP assay (Figure 2*A*, dark blue).

We further characterized the SOD1 aggregates using detergent solubility. The large molecular weight material was resistant to non-ionic detergent (0.5% Triton X-100), but was readily dissociated by 5% SDS at room temperature (Figure 2*D*).

Thus, mutant SOD1 in *C. elegans* appears to form a molecularly heterogeneous mixture of SDS-labile aggregates and soluble protein, which for G85R and 127X does not attain a stably folded, native conformation.

### Mutant SOD1 Aggregates Display Morphological Heterogeneity

**Figure 3:**
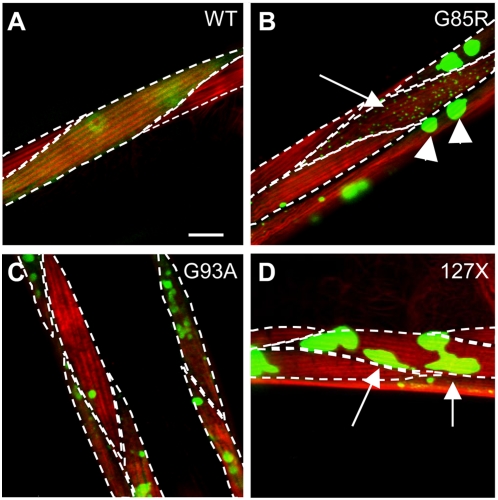
Mutant SOD1 forms morphologically distinct aggregates. Projections of confocal Z-stacks with several adjacent muscle cells; punctate lines delineate individual cells, red staining shows myofilaments stained with Rhodamine-phalloidin. G85R protein (*B*) forms morphologically diverse aggregates, including large foci indicated by arrowheads and small dispersed foci, indicated by arrow. Aggregates in about 40% of cells of 127X animals appear as irregular, elongated foci (*D*, arrows and inserts in Figure 1 *J–L*). Scale bar in *A* is 10 micrometers. Images are selected as representative of typical aggregation morphology among at least 100 imaged cells per genotype.

The observed biochemical heterogeneity paralleled a striking heterogeneity in aggregate morphology and distribution in SOD1-transgenic strains. While all three mutant SOD1 strains had some cells devoid of visible aggregates, most contained aggregates that exhibited a wide range of shapes, sizes, and cellular distribution (Figure 3). The majority of cells (up to 75%) in G85R animals contained 1–5 aggregates with the apparent size of the fluorescent foci of 5–7 µm, while some cells contained more than 20 smaller (less than 1 µm) dispersed aggregates (Figure 3*B*). Both types of aggregates exhibited similar biophysical properties by FRAP analysis (not shown). The G93A strain had a more uniform distribution of morphological types (Figure 3*C*), with aggregates often seen in close apposition to each other (Figure S4*A,B*). 127X animals differed from both G85R and G93A strains in that they contained up to 40% of cells with irregular, non-spherical aggregates (Figure 3*D*). The presence of distinct morphological classes did not depend on the expression level, as we observed a similar distribution in heterozygous SOD1 animals, despite lower extent of aggregation (not shown). These data show that the wild type and three different mutants of SOD1 form morphologically, structurally and enzymatically different molecular species *in vivo*, supporting the possibility of distinct interactions with cellular components.

### Expression of Mutant SOD1 Causes Limited Toxicity

**Figure 4:**
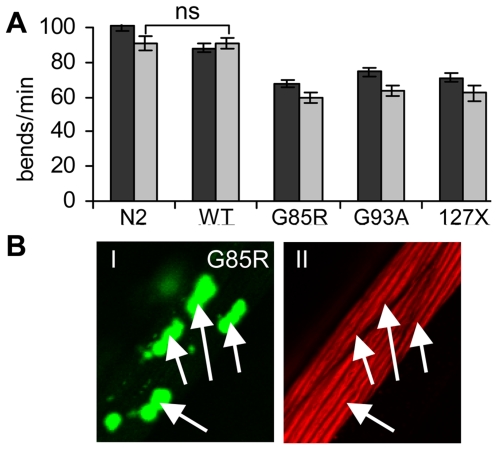
SOD1 mutants exert mild toxicity in the body-wall muscle cells. (*A*) Body bends of individual animals per minute were measured. Expression of mutant SOD1 proteins lead to 25 to 30% decrease in motility on day 2 of adulthood (dark grey bars), and further decrease by approximately 10% on day 6 of adulthood (light grey bars). Error bars are standard error of the mean, n≥15. *P*<0.001 between N2 and each SOD1 strain, except where indicated (ns). (*B*) SOD1 aggregation does not disrupt the myofilaments structure. Panel *I* shows distribution of G85R aggregates in several muscle cells, panel *II* shows Rhodamine-phalloidin staining of filaments in same cells. Both panels represent a single confocal plane with aggregates (*I*) or filaments (*II*) in focus.

We next asked whether expression of these aggregation-prone proteins caused toxicity. We assessed several phenotypes as indicators of dysfunction of muscle cells expressing the transgenes, such as decrease in motility of animals, disturbance of ultrastructural organization of myofilaments, developmental defects, and egg-laying defects. The motility of WT SOD1 animals grown at 15°C was similar to that of wild type (N2) strain on the second day of adulthood, as measured by number of body bends per minute (Figure 4*A*). In contrast, animals expressing G85R, G93A or 127X SOD1 mutant proteins had 25–30% reduction in motility relative to that of N2 animals. This decrease in motility was only minimally enhanced by the sixth day of adulthood (Figure 4*A*, light grey bars).

To assess the ultrastructural organization of myofilaments, we visualized actin filaments with Rhodamine-labeled phalloidin. We found no major disruptions of the organization of actin filaments in cells containing aggregates in either of three mutant strains (Figures 4*B*, S4*A*). In fact, SOD1 aggregates were localized to a different focal plane than the myofilaments (Figure S4*B*), unlike polyQ aggregates, which were found intercalating into filaments and disrupting their structure (Figure S4 *AVII* and *AVIII*).

Dysfunction of muscle cells during embryonic development leads to defective elongation of the body shape of *C. elegans* and to embryonic lethality (*emb*) or hatching of deformed, growth arrested larvae (*lva*). Although SOD1 mutant proteins aggregate already in embryos, neither of the mutant SOD1 strains exhibited substantially elevated *emb+lva* phenotype relative to the WT SOD1 strain (Table 1). The highest toxicity was in G85R strain (8.2% phenotype, compared to 1.8% in WT SOD1). Likewise, despite the presence of aggregates in vulva muscle cells, no increase in egg laying defect was found in mutant SOD1 strains (Table 1) compared with WT SOD1 strain. Thus, under given experimental conditions, expression of mutant SOD1 proteins seems to exert limited toxicity in the muscle cells of *C. elegans*.

**Table 1:** SOD1 toxicity is modulated by destabilizing protein polymorphisms.

		15°C					25°C	
phenotype	protein expressed	SOD1	param(ts)	Ras(ts)	SOD+param(ts)	SOD+Ras(ts)	param(ts)	Ras(ts)
*emb+lva*	WT SOD	1.8+/−1.2			0.9+/−1.6	**		
	G85R	8.2+/−2.5			55.1+/−12.8	7.1+/−8.0		
	G93A	3.8+/−1.6			44.4+/−8.8	10.6+/−2.8		
	127X	0.3+/−0.4			33.8+/−9.5	23.3+/−4.0		
	param(ts)		6.8+/−0.1				100+/−0.0	
	Ras(ts)			3.5+/−2.7				100+/−0.0
**phenotype**	**protein expressed**	**SOD1**	**UNC-45(ts)**	**Ras(ts)**	**SOD+UNC-45(ts)**	**SOD+Ras(ts)**	**param(ts)**	**Ras(ts)**
*egl+rep*	WT SOD	6.3+/−4.6			9.5+/−3.0	**		
	G85R	7.1+/−2.1			64.8+/−6.6	14.4+/−9.2		
	G93A	3.4+/−1.8			81.0+/−8.5	30.9+/−8.3		
	127X	5.5+/−3.9			54.6+/−17.3	72.3+/−12.0		
	UNC-45(ts)		3.4+/−0.1				100+/−0.0	
	Ras(ts)			6.9+/−4.0				93.1+/−7.0

**: - these phenotypes were not measured due to inability to obtain double homozygous animals.

### The Toxicity of Mutant SOD1 in Muscle Cells Is Modulated and Directed by Destabilizing Protein Polymorphisms

The relatively mild toxicity, despite misfolding and aggregation of mutant SOD1, indicates that the putative toxic species are either transient or suppressed by the cellular folding/quality control machinery. We had previously found that metastable temperature-sensitive (ts) mutations in various unrelated genes, such as *unc-15*, *unc-45* and *let-60*, coding for paramyosin(ts), UNC-45(ts) and Ras(ts) proteins, respectively, destabilized the cellular folding environment and modulated the toxicity of polyQ expansions [35]. To examine whether protein polymorphisms in genetic background could influence SOD1 toxicity, we introduced WT and mutant SOD1 proteins into ts mutant strains and assayed ts phenotypes at the permissive temperature. WT SOD1 showed no synthetic toxicity with ts mutants. In contrast, G85R, G93A and 127X mutants of SOD1 caused exposure of each specific ts phenotypes at permissive temperature (Table 1).

Loss of function of paramyosin during embryonic development leads to defects in muscle structure and thus to the *emb+lva* phenotype [36]. Expression of mutant SOD1 in the paramyosin(ts) strain caused differential exposure of these phenotypes at the permissive temperature (Table 1), depending on the identity of the SOD1 mutant: expression of G85R resulted in 55% of *emb+lva* at 15°C, 127X had 33% of *emb+lva* phenotype, and G93A had intermediate toxicity. The surviving animals had very few progeny. To ask whether this toxic interaction is specific to paramyosin, we crossed SOD1 strains to a strain harboring a ts mutation in *unc-45* gene. Expression of SOD1 mutant proteins in *unc-45(ts)* genetic background resulted in exposure of egg laying and reproductive phenotype (*egl+rep*) at the permissive temperature (Table 1). This phenotype is characteristic of dysfunction of UNC-45-expressing embryonic muscle cells, vulva muscle cells and gonad sheath cells, and is present in a 100% of *unc-45(ts)* mutant animals at the restrictive temperature. Here, G93A exhibited over 80% toxicity at 15°C, compared with less than 5% for either G93A or UNC-45(ts) expressed alone. Furthermore, the surviving animals expressing both G93A and UNC-45(ts) developed into severely uncoordinated (Figure S5), sick adults.

Paramyosin and UNC-45 both affect the formation of myofilaments: paramyosin is a structural component and UNC-45 regulates myosin assembly. To assess whether SOD1 mutants were toxic towards a metastable mutant in a different cellular pathway, we crossed SOD1 strains to a strain expressing a temperature-sensitive Ras variant. Expression of G85R and G93A in *ras(ts)* background did not have strong effects on embryonic lethality (Table 1), while 127X, which was the least toxic with paramyosin(ts) and UNC-45(ts), caused lethality in 23% of embryos. SOD1 mutant proteins did cause Ras(ts) animals to exhibit defects in egg laying (Table 1), with 72% *egl+rep* phenotype exposed in 127X and 14% in G85R animals. The ts strain by itself, however, did not present the same egg laying defect (*egl*) at 25°C. Instead, Ras(ts) animals raised at the restrictive temperature had a fluid-filled appearance with degenerated gonads, and produced few embryos (100% reproductive phenotype, *rep*). Note that Table 1 shows a combined *egl+rep* phenotype. Ras is ubiquitously expressed, and pleiotropic phenotypes that are exposed at the restrictive temperature reflect its dysfunction in different cell types. The *egl* phenotype exposed by SOD1mutants in *ras(ts)* background is likely due to the genetic interaction between the two mutations specifically in the in vulva muscles or gonad sheath cells, where the *Unc-54* promoter driving SOD1 expression is active.

These data show that while expression of the three distinct SOD1 mutants in the wild-type N2 strain of *C. elegans* leads to mild toxicity, their expression in the genetic backgrounds harboring diverse temperature-sensitive mutations uncovers toxic phenotypes, with each SOD1 mutant affecting the activity of a given metastable protein to different extents.

## Discussion

We show here that introduction of mildly destabilized protein polymorphisms into the defined genetic background of *C. elegans* modulates the toxicity of three mutant SOD1 proteins, leading to the development of specific toxic phenotypes. While either SOD1 aggregation or loss-of-function of metastable ts proteins can each be viewed as a separate consequence of failure of protein folding homeostasis, the toxic phenotypes observed here resulted from their genetic interaction and thus were directed by the nature of the ts mutation present. Similar to ts mutations in *C. elegans*, the phenotypic expression of mildly destabilizing protein polymorphisms in higher organisms is thought to depend on the robustness of the protein folding environment [37]. Thus, the demand on the folding resources as a consequence of aging, proteotoxic conditions, and genetic background, may alter the threshold for the toxicity of an aggregation-prone protein, while specific pathways and protein complexes containing such polymorphisms may direct cell-type specific phenotypes.

### *C. elegans* Model for SOD1 Aggregation and Toxicity

We established a *C. elegans* model in which aggregation, toxicity, and cellular interactions can be directly compared between different SOD1 mutants. Furthermore, as models of other aggregation-prone proteins, such as polyglutamine expansions [25], α-synuclein [27], and Aβ [38] use similar expression schemes in the same N2 genetic background, these *C. elegans* models could be instrumental in deciphering both common and protein-specific regulation of aggregation or toxicity. Expression of three different ALS-related mutants of SOD1 in body-wall muscle cells of *C. elegans* lead to mild cellular disfunction and appearance of protein aggregates with distinct morphological characteristics. We also observed an unexpected variability of aggregate morphology in neighboring cells of the same animal, which could indicate that factors other than genetically encoded interactions also affect the fate of SOD1 in the cell. Similar stochastic variability between muscle cells of the same animal was previously reported with respect to onset of sarcopenia in wild type *C. elegans* [39].

We find that ALS-related SOD1 variants exert a potent destabilizing influence on the functionality of metastable temperature-sensitive proteins at permissive conditions, exposing a range of phenotypes that are not present in strains expressing SOD1 mutants alone. Moreover, the most toxic of the SOD1 mutants (127X) in the Ras(ts) background was the least toxic in the paramyosin(ts) background, whereas the G85R mutation was most toxic with paramyosin(ts). Strain-dependent differences in SOD1 toxicity were previously observed in a mouse model carrying G86R mutation in murine *SOD1* (corresponding to G85R in human *SOD1*), with complete suppression of toxicity up to 2.5 years in one genetic background, but a rapid onset of paralysis by 90–120 days in a different genetic background [23]. Our data suggests that mildly destabilizing missense mutations, present in the genetic background, could effect the exposure of specific phenotypes.

### Disruption of Protein Folding Is a Common Mechanism of Toxicity of Aggregation-Prone Proteins

The nature of the toxicity of aggregation-prone proteins remains one of the central questions for diseases of protein conformation. We have previously showed that unrelated ts mutations caused premature aggregation of polyQ-expanded proteins [35]. Furthermore, metastable proteins encoded by these ts mutations were found to misfold and lose function in polyQ strains, indicating that protein folding homeostasis was disrupted by chronic protein misfolding. Unlike polyQ, mutant SOD1 proteins, though highly aggregation-prone, exhibited much lower toxicity on their own. The demonstration that toxicity of both mutant SOD1 and polyQ expansions can be modulated by metastable proteins supports our contention that the proteostasis network [40] is sensitive to cumulative protein damage, and that the disruption of protein folding may be a common mechanism that underlies the toxicity of different aggregation-prone proteins.

### Aggregation-Prone Proteins and Mildly Destabilized Protein Polymorphisms Compete for Folding Resources

Each of the SOD1 mutant proteins used in this study exhibits distinct biophysical properties *in vitro* [28], and forms morphologically, structurally and enzymatically distinct molecular species and aggregates in *C. elegans*. It is thus possible that SOD1 mutant proteins form different intermediate folding states *in vivo* depending on the nature of the mutation, and as such may possess different functional interactions with the folding machinery of the cell. Indeed, G85R and G93A proteins were recently shown to have different interactions with HSP70 in cultured cells [41]. On the other hand, the structure and functions of paramyosin, UNC-45, and Ras are diverse (a structural coiled-coil protein, a soluble TPR domain-containing protein and a small GTPase, respectively) and these proteins are not overexpressed as they are expressed from their endogenous chromosomal loci. Thus, it is unlikely that the synergistic effects on toxicity are because of direct and specific molecular interactions between these protein polymorphisms and mutant SOD1. This is in agreement with our previous observation that polyQs cause misfolding of metastable proteins in the absence of direct molecular interactions [35], and with a recent report that many of the modifiers of toxicity of polyQ-expanded ataxin-3 in *Drosophila* also rescue the generic toxicity of protein misfolding due to the reduced function of HSP70 [42]. Furthermore, both the functionality of metastable proteins and polyglutamine aggregation can be compromised by neuronally-mediated overexcitation of the muscle cells in *C. elegans* [43]. These findings parallel recent computational evidence that the selection against the toxicity of misfolding due to mistranslation exerts strong evolutionary pressure specifically on the highly expressed proteins [44], indicating that the flux of destabilized proteins in a cell bears a significant fitness cost, and that folding resources are indeed limiting. In support of this, we show that overexpression of the heat-shock transcription factor HSF-1 rescues the toxic phenotypes in a strain co-expressing an SOD1 mutant G93A and a metastable ts mutant of UNC-45 (Figure S5*B*). Thus, we propose that the genetic interactions between disease-causing mutations and mildly destabilizing protein polymorphisms are mediated at the cellular level by competition of their respective gene products for folding resources.

### Cell-Type Specific Toxicity of Aggregation-Prone Proteins

This hypothesis could offer an explanation for the apparent paradox of cell-type-specific toxicity caused by ubiquitously expressed toxic proteins in conformational diseases. Indeed, in SOD1-related ALS, Huntington's disease, and Alzheimer's disease, specific neuronal subtypes are affected despite ubiquitous expression of SOD1, huntingtin and APP, respectively. The differential modulation of mutant SOD1 toxicity in *C. elegans* by specific ts mutations suggest that the presence of mildly destabilizing protein polymorphisms in the genetically diverse human population could direct such specific phenotypes: because each cell type contains characteristic complement of expressed proteins, the genetic interactions of aggregation-prone proteins with destabilizing polymorphisms are expected to manifest in a cell type-specific manner. The disease variability across the population suggests that such protein polymorphisms may be specific to individuals or families, and missed in the population-based linkage analyses. A recent study found that up to 70% of rare missense alleles are mildly deleterious in humans [45]; some of these polymorphisms may result in the production of metastable or folding-deficient proteins [46],[47]. Identification of cell-specific pathways or protein complexes, which may disfunction when folding or stability of their components is challenged by co-expression with an aggregation-prone protein, may thus provide specific toxic mechanisms for conformational diseases and help focus the search for disease-modifying polymorphisms.

## Materials and Methods

### Nematode Strains and Growth Conditions

Nematodes were grown on NGM plates seeded with E. coli OP50 strain. Animals were synchronized by picking L4 larva or pre-comma stage embryos onto fresh plates. Assays were performed with young adult animals, at the second day of reproductive adulthood at either 15°C (3.5 days after L4 stage) or 25°C (2 day after L4 stage). *C. elegans* strains were obtained from the Caenorhabditis Genetic Center. Ts mutants were: paramyosin(ts) - CB1402[*unc-159(e1402)*], UNC-45(ts) - CB286[*unc-45(e286)*] and Ras(ts) - SD551[*let-60(ga89)*].

The SOD1 transgenic strains were created by injection and integration of complex arrays, allowing for uniform expression of transgenes. Human SOD1 sequences were obtained by PCR amplification from plQL01 or plQL03 (gift from Dr. Q. Liu, Harvard Medical School), and cloned into a Fire Lab pPD30.38 plasmid. DNA mixture for injection contained 1 ng of linearized plasmid DNA and 100 ng of worm genomic DNA digested with PvuII (NEB).

Crosses between SOD1 transgenic and ts strains were performed by first mating N2 males with SOD1 hermaphrodites, and subsequently mating SOD1 heterozygous fluorescent males with ts hermaphrodites. 3–5 fluorescent F1 hermaphrodite progeny from these crosses were allowed to self, and 15–20 F2 fluorescent progeny were singled onto individual plates. Plates containing 100% temperature-sensitive progeny were used for generation of double-homozygous strains. To generate a strain double homozygous for G85R and Ras(ts), a singe fluorescent F2 hermaphrodite exhibiting a strong *muv* phenotype was picked directly from a pool of F2 progeny. We could not generate a WT SOD+Ras(ts) animals, presumably due to close genetic location of respective loci.

We noted that strains co-expressing SOD1 and ts mutant proteins tended to accumulate suppressors, similar to what was observed with polyQ expansions [35]. Strains with low progeny number were particularly unstable, resulting in segregation of strains revertant for the toxic phenotype either during selection of homozygotes or in the first few generations thereafter, or in gradual improvement of fitness of the entire population (data from such strains were discarded). All double homozygous lines were periodically re-built, and assays were performed within two weeks of obtaining double homozygous animals.

### Biophysical Characterization and Enzymatic Assay

Fluorescence recovery after photobleaching (FRAP) analysis was performed as previously described [48]. Imaging was performed on a Zeiss LSM 510 Meta confocal microscope. YFP alone (polyQ0) and polyQ40 strains, used as controls for soluble and stably aggregated protein species, are described in [34].

For native extracts, nematode pellets were mechanically disrupted, lysed in native lysis buffer (50 mM Tris pH7.4, 5 mM MgCl_2_, 0.5% Triton-X 100, 0.2 mM PMSF, 1 ug/ml Leupeptin, 1 ug/ml Pepstatin A, Complete protease inhibitor (Roche)) and centrifuged for 1 min at 30×g (Eppendorf 5417C centrifuge). All reagents were from Sigma, unless indicated otherwise. This protocol is optimized for the removal of debris and large fragments of cuticle while preserving the majority of aggregates in the supernatant, verified by examination of supernatant under fluorescent microscope. For detergent solubility, native extracts were incubated in the indicated detergent for 15 min at room temperature prior to resolving by native PAGE. 20 or 30 micrograms total protein was run on a 5% or 7.5% continuous native gels. Gels were imaged on Storm 860 scanner (Molecular Dynamics) with ImageQuant software to detect YFP fluorescence, or processed for the in-gel enzymatic assay.

### Immunostaining and Microscopy

For epifluorescence, nematodes were mounted on 1% agarose pads with 1 mM levamisole and imaged using Zeiss Axiovert 200 microscope. For immunofluorescence and confocal imaging, synchronized adults were fixed, permeabilized and stained with Rhodamine-phalloidin (Molecular Probes), as described previously [35], and imaged with Zeiss LSM 510 Meta confocal microscope through a 40×1.0 numerical aperture objective with either a 514-nm or 543-nm line for excitation.

### Motility Assay

To measure motility, nematodes at indicated age were placed individually in a drop of M9 buffer and acclimated for 1 min; the completed body bends were counted for 1 min. At least15 animals were used per experiment. Similar decrease in motility was found by this method and by measuring rate of movement of animals raised at 20°C on a plate seeded with OP50 bacteria (not shown).

### Assays for Specific Temperature-Sensitive Phenotypes

For *emb*+*lva* at 15°C, freshly laid pre-comma stage embryos were picked onto new plates. Unhatched embryos and larvae that hatched but did not crawl or were severely deformed were scored after 2 days. Alternatively, young adults were acclimated to 25°C for 1 day prior to egg laying, transferred onto new plates and allowed to lay embryos. Embryos were picked and scored one day later at 25°C. About one hundred embryos was used per experiment and experiments were repeated at least three times.

To score *egl*+*rep* and severe uncoordination, 30 L4 larvae grown at 15°C were picked to a fresh plate and incubated for 3 days at 15°C or 1.5 days at 25°C. Animals retaining eggs or containing three-fold embryos (detected with Nomarski optics) were scored as egg laying defective (*egl*). Animals with degenerated gonads, sterile, and those accumulating oocytes were scored as having a reproductive defect (*rep*). Animals that did not move on their own or did not exhibit sinusoidal movement pattern after being prodded were scored as severely uncoordinated. Experiments were repeated at least three times.

## Supporting Information

Figure S1 (A) Schematic representation of the SOD1-YFP expression constructs. The *Unc-54* promoter/enhancer and 3′UTR direct expression of the fusion protein in body wall, intestinal, anal depressor, and sphincter muscles, as well as sex-specific muscles that develop postembryonically (WormBase). (B) Steady-state protein levels of SOD1 WT and mutant proteins. G85R and 127X proteins are expressed at level similar to the WT SOD1, while G93A is expressed at lower steady-state level. The level of YFP protein in the control strain is more than 2 fold higher than in any of the SOD1-YFP strains. The upper panel shows immunoblot with anti-YFP antibody, the bottom panel - with anti-tubulin antibody. 10 individual young adult animals were picked from indicated strains, boiled (15 min) in SDS sample buffer and resolved on 10% SDS gel. Immunoblots were scanned and quantified using Odyssey Infrared Imaging System (LI-COR Biosciences). The numbers below the gel represent quantitation of YFP signal normalized to tubulin.
(0.53 MB TIF)

Figure S2 Only soluble species of WT SOD1 and G93A proteins possess specific dismutase activity. G93A extract (line 3, arrow) contains one main population of similar electrophoretic mobility (A) and enzymatic activity (B) to the WT SOD1 (line 1, arrow). The aggregated G93A protein is inactive (arrowhead), as is G85R and 127X protein (lanes 2 and 4, respectively). Native extracts were resolved by 5% native PAGE (same gel as in Figure 2D, upper panel), YFP fluorescence was visualized with Storm 860 scanner, and in-gel enzymatic activity assay was subsequently performed as previously described [49]. The assay measures SOD1-mediated inhibition of nitro blue tetrazolium (NBT) reduction by riboflavin and TEMED. 20 micrograms total protein was used for this assay.
(1.34 MB TIF)

Figure S3 The aggregation pattern and morphological variability of aggregates in G85R strain. (A) Aggregation pattern of G85R mutant protein at 20°C is similar to that observed at 15°C (shown in Figure 1H). Shown are Nomarski and fluorescent micrographs of representative L4 WT SOD1 and G85R animals. The scale bar in panel I is 50 micrometers. (B) Area posterior to the vulva is shown in four individual young adult G85R animals grown at 15°C. The nematodes were anesthetized, but not fixed, prior to microscopic examination.
(3.56 MB TIF)

Figure S4 SOD1 aggregates do not disrupt myofilaments and localize to the muscle belly. (A) Phalloidin-stained myofilaments (red) appear intact in the cells containing SOD1 aggregates (green, panels I through VI). In contrast, polyQ40 aggregates (panel VII) intercalate into myofilaments and disrupts their continuity (panel VIII). Arrows in each panel point to the location of selected aggregates. Panels I, III and V, showing SOD1 aggregates, and II, IV and VI, showing myofilaments in corresponding cells, are in different confocal planes. The scale bar in I is 10 micrometers. (B) G93A aggregates are localized to the cytoplasmic area beneath the myofilaments - muscle belly. Confocal image through the middle plane of individual nematode, the muscle cells on the ventral side (towards the bottom of the image) are seen edge-on. Short arrows indicate positions of the muscle quadrants, arrowheads outline an oocyte. Several G93A aggregates (green) are seen in close apposition to each other (long arrow), adjacent to myofilaments (red). The scale bar is 10 micrometers.
(3.06 MB TIF)

Figure S5 Expression of SOD1 mutants in *unc-45(ts)* background leads to defect in cellular protein folding and exposure of severe uncoordination phenotype. (A) Double homozygous animals were scored on day 2 of adulthood. Animals that did not move on their own or did not exhibit sinusoidal movement pattern after being prodded were scored as severely uncoordinated. The hatched bar represents *unc-45(ts)* animals at 25°C. (B) Overexpression of heat-shock transcription factor HSF-1 rescues the synergistic toxicity between G93A and UNC-45(ts) mutant proteins. HSF-1 is expressed from ubiquitous promoter *let-858*, as described in [50].
(0.35 MB TIF)
