# Supplementary figures and images for: Destabilizing Protein Polymorphisms in the Genetic Background Direct Phenotypic Expression of Mutant SOD1 Toxicity
Source: PLoS Genet. 2009 Mar 6;5(3):e1000399. doi: 10.1371/journal.pgen.1000399 (PMC2642731; doi:10.1371/journal.pgen.1000399)

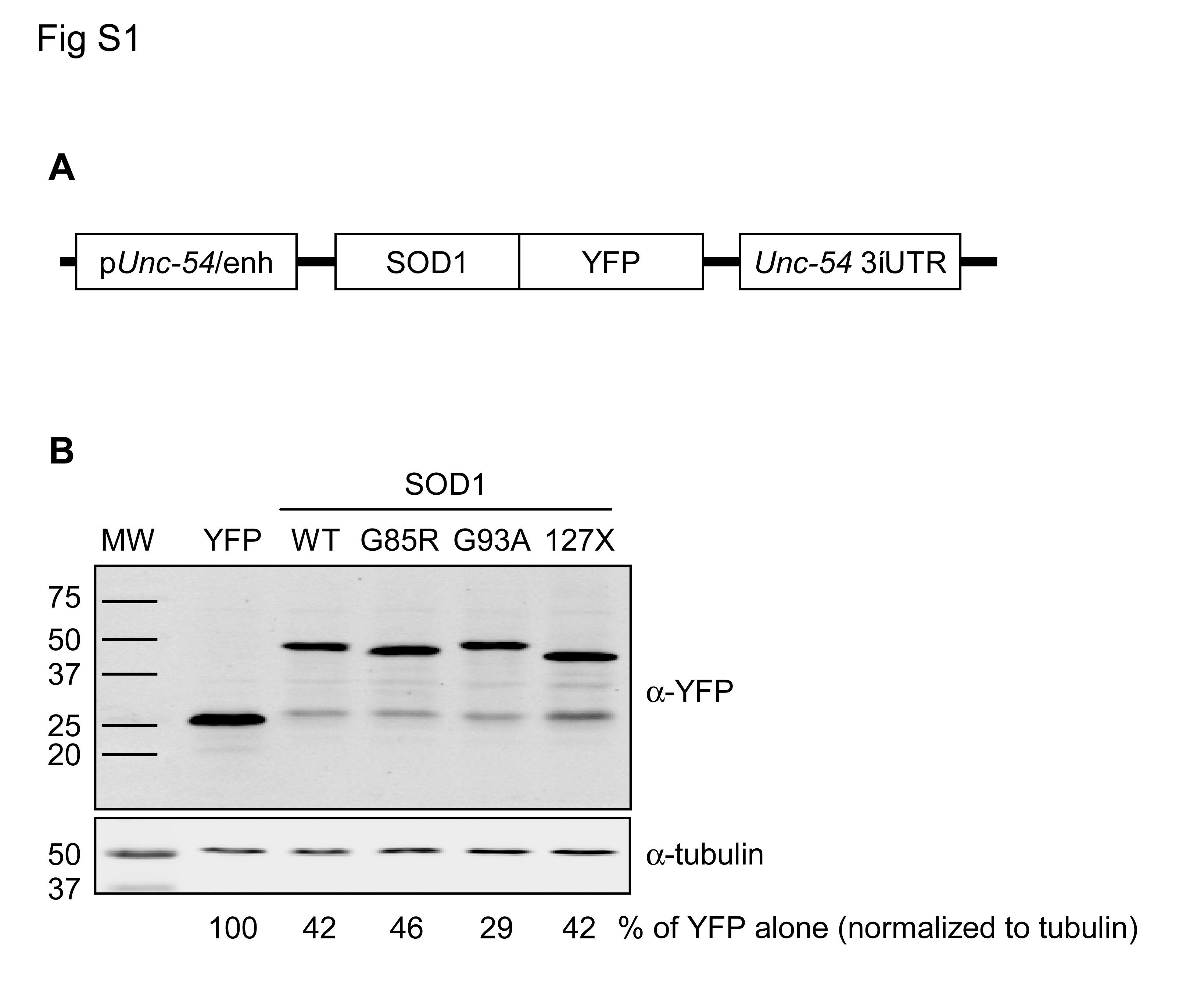

Supplement: Figure S1 — (A) Schematic representation of the SOD1-YFP expression constructs. The Unc-54 promoter/enhancer and 3′UTR direct expression of the fusion protein in body wall, intestinal, anal depressor, and sphincter muscles, as well as sex-specific muscles that develop postembryonically (WormBase). (B) Steady-state protein levels of SOD1 WT and mutant proteins. G85R and 127X proteins are expressed at level similar to the WT SOD1, while G93A is expressed at lower steady-state level. The level of YFP protein in the control strain is more than 2 fold higher than in any of the SOD1-YFP strains. The upper panel shows immunoblot with anti-YFP antibody, the bottom panel - with anti-tubulin antibody. 10 individual young adult animals were picked from indicated strains, boiled (15 min) in SDS sample buffer and resolved on 10% SDS gel. Immunoblots were scanned and quantified using Odyssey Infrared Imaging System (LI-COR Biosciences). The numbers below the gel represent quantitation of YFP signal normalized to tubulin. (0.53 MB TIF) [file pgen.1000399.s001.tif]

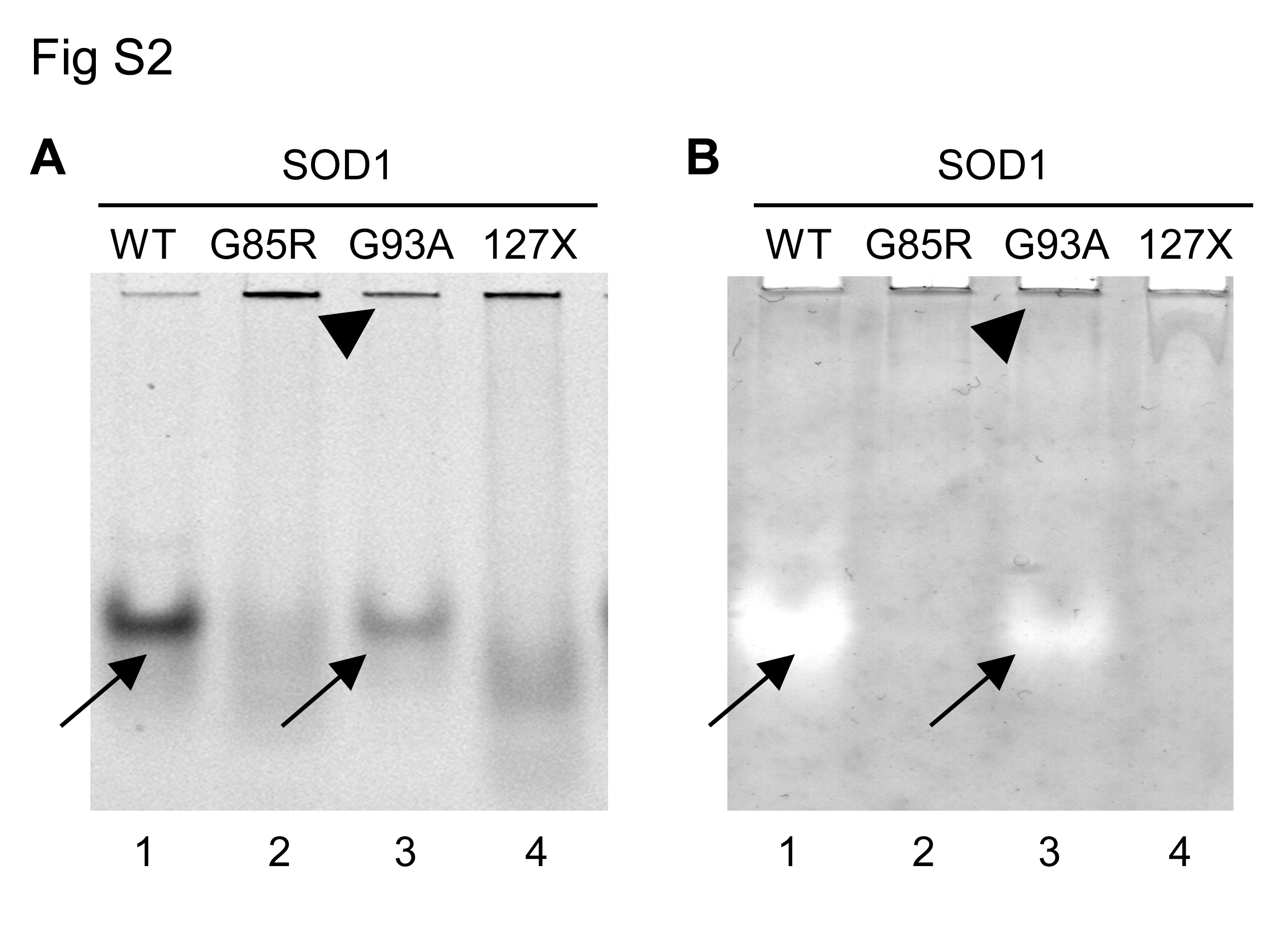

Supplement: Figure S2 — Only soluble species of WT SOD1 and G93A proteins possess specific dismutase activity. G93A extract (line 3, arrow) contains one main population of similar electrophoretic mobility (A) and enzymatic activity (B) to the WT SOD1 (line 1, arrow). The aggregated G93A protein is inactive (arrowhead), as is G85R and 127X protein (lanes 2 and 4, respectively). Native extracts were resolved by 5% native PAGE (same gel as in Figure 2D, upper panel), YFP fluorescence was visualized with Storm 860 scanner, and in-gel enzymatic activity assay was subsequently performed as previously described [49]. The assay measures SOD1-mediated inhibition of nitro blue tetrazolium (NBT) reduction by riboflavin and TEMED. 20 micrograms total protein was used for this assay. (1.34 MB TIF) [file pgen.1000399.s002.tif]

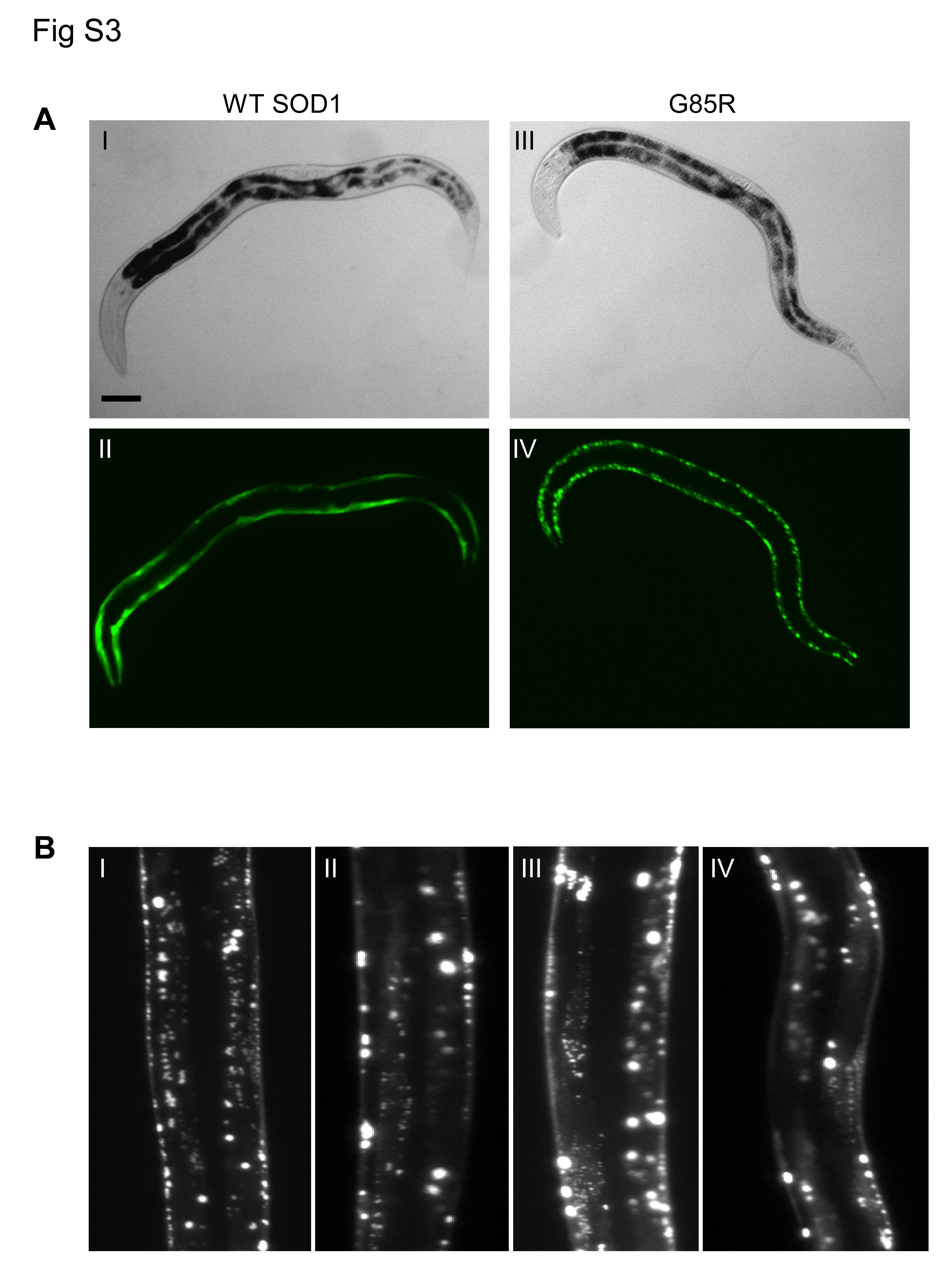

Supplement: Figure S3 — The aggregation pattern and morphological variability of aggregates in G85R strain. (A) Aggregation pattern of G85R mutant protein at 20°C is similar to that observed at 15°C (shown in Figure 1H). Shown are Nomarski and fluorescent micrographs of representative L4 WT SOD1 and G85R animals. The scale bar in panel I is 50 micrometers. (B) Area posterior to the vulva is shown in four individual young adult G85R animals grown at 15°C. The nematodes were anesthetized, but not fixed, prior to microscopic examination. (3.56 MB TIF) [file pgen.1000399.s003.tif]

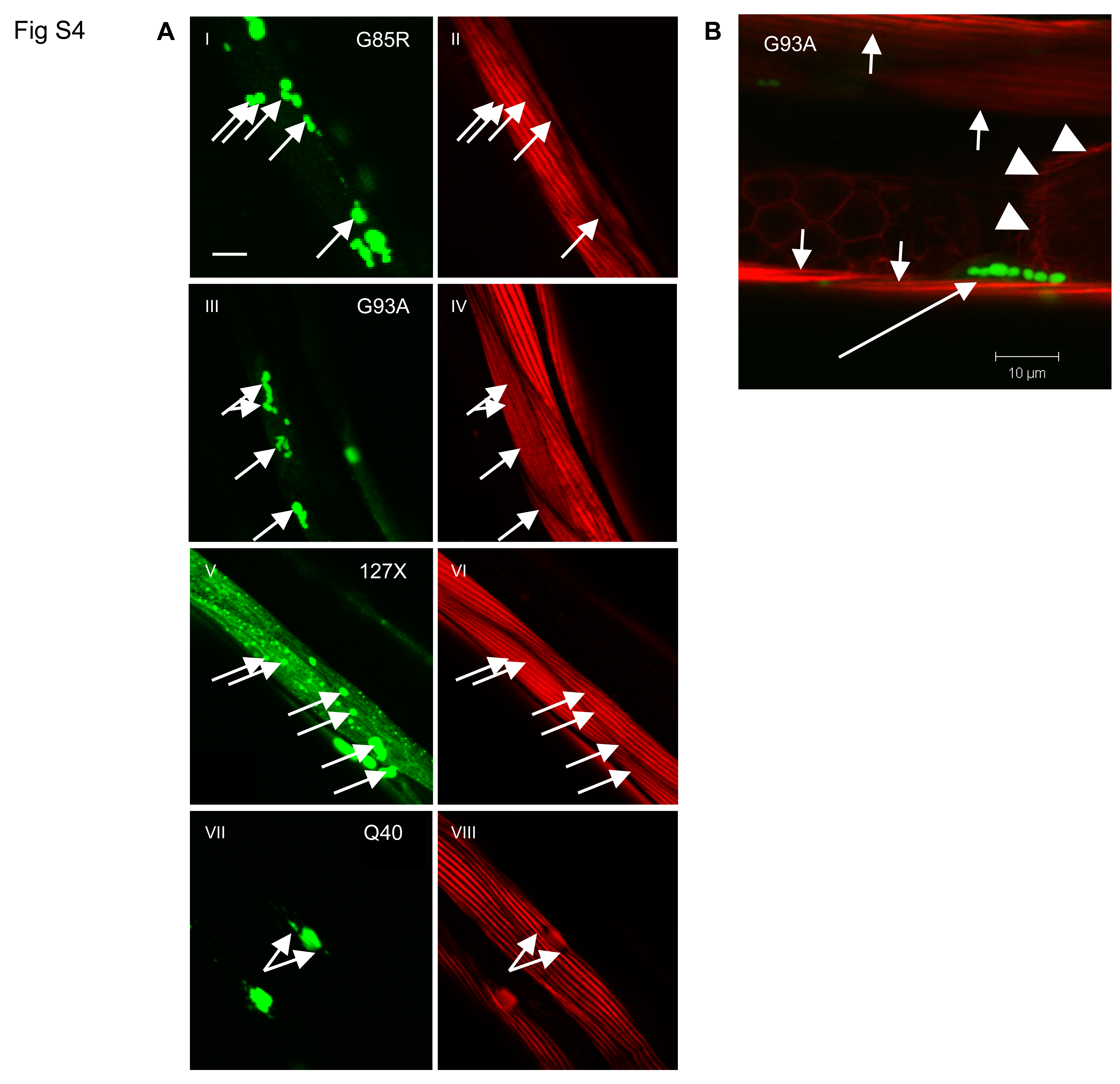

Supplement: Figure S4 — SOD1 aggregates do not disrupt myofilaments and localize to the muscle belly. (A) Phalloidin-stained myofilaments (red) appear intact in the cells containing SOD1 aggregates (green, panels I through VI). In contrast, polyQ40 aggregates (panel VII) intercalate into myofilaments and disrupts their continuity (panel VIII). Arrows in each panel point to the location of selected aggregates. Panels I, III and V, showing SOD1 aggregates, and II, IV and VI, showing myofilaments in corresponding cells, are in different confocal planes. The scale bar in I is 10 micrometers. (B) G93A aggregates are localized to the cytoplasmic area beneath the myofilaments - muscle belly. Confocal image through the middle plane of individual nematode, the muscle cells on the ventral side (towards the bottom of the image) are seen edge-on. Short arrows indicate positions of the muscle quadrants, arrowheads outline an oocyte. Several G93A aggregates (green) are seen in close apposition to each other (long arrow), adjacent to myofilaments (red). The scale bar is 10 micrometers. (3.06 MB TIF) [file pgen.1000399.s004.tif]

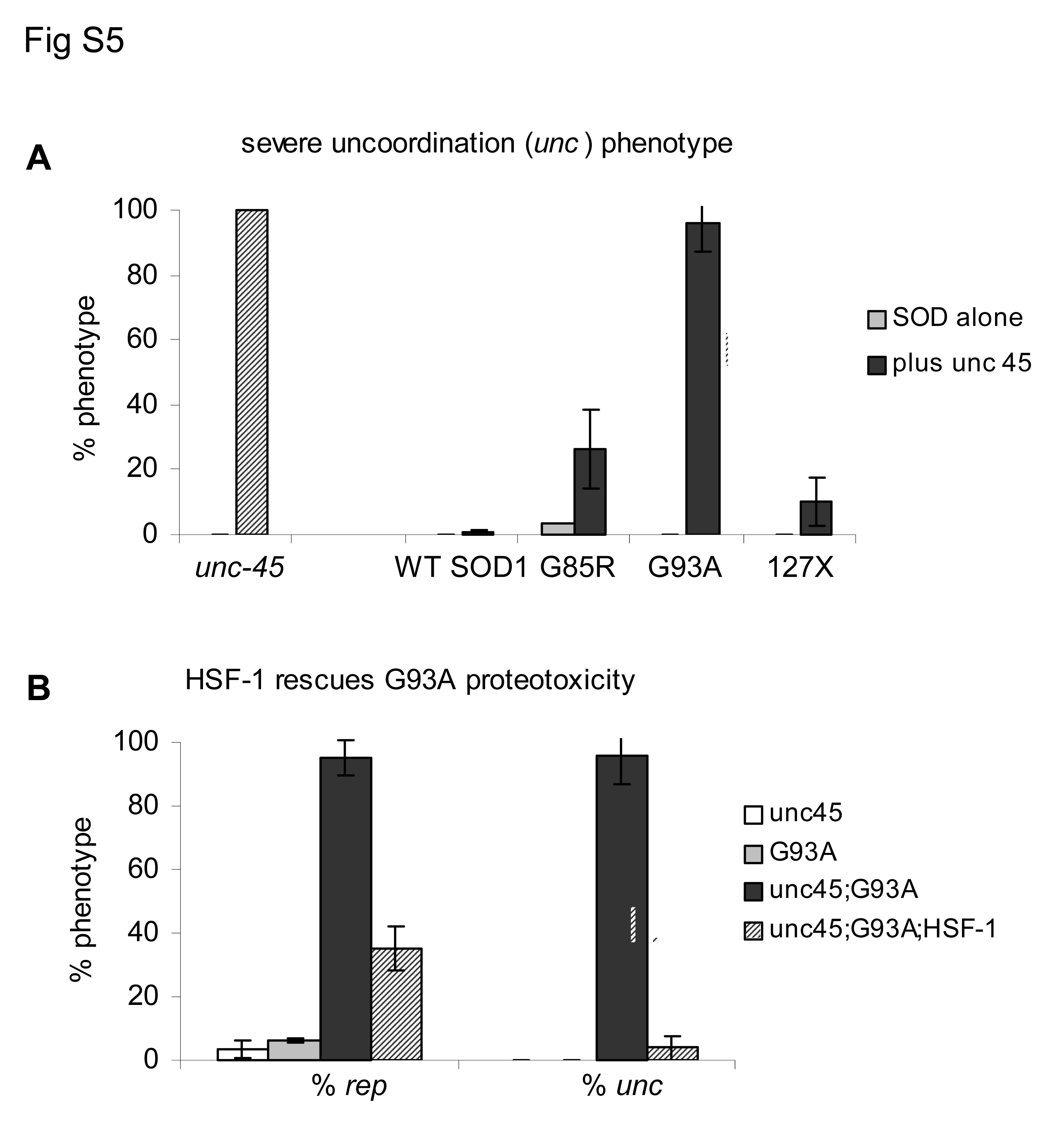

Supplement: Figure S5 — Expression of SOD1 mutants in unc-45(ts) background leads to defect in cellular protein folding and exposure of severe uncoordination phenotype. (A) Double homozygous animals were scored on day 2 of adulthood. Animals that did not move on their own or did not exhibit sinusoidal movement pattern after being prodded were scored as severely uncoordinated. The hatched bar represents unc-45(ts) animals at 25°C. (B) Overexpression of heat-shock transcription factor HSF-1 rescues the synergistic toxicity between G93A and UNC-45(ts) mutant proteins. HSF-1 is expressed from ubiquitous promoter let-858, as described in [50]. (0.35 MB TIF) [file pgen.1000399.s005.tif]
